# 580 Setting the Burn Team Up for Success

**DOI:** 10.1093/jbcr/irac012.208

**Published:** 2022-03-23

**Authors:** Cassandra O'Rourke, Megan Gernt

**Affiliations:** Rhode Island Burn Center, Providence, Rhode Island; Rhode Island Hospital, Attleboro, Massachusetts

## Abstract

**Introduction:**

Hybrid burn centers are challenged to meet the criteria of the ABA verification process. When burn care occurs in different units across a hospital, it is difficult to ensure all areas are aware of the verification criteria and ensure timelines and expectations of optimal burn care are understood. Our center had difficulty ensuring rehabilitation referrals were placed on admission to allow the maximum amount of time for these services to establish a plan of care. Staffing issues were identified as contributing to the inability to consistently establish a rehabilitation program within 24 hours of admission.

**Methods:**

We had a QI goal of 100% compliance for establishing a comprehensive rehabilitation plan within 24 hours of admission. Several methods of education were provided to all providers and other burn team members to encourage immediate consultation to physical therapy and occupational therapy. Consultations to these services were frequently delayed. In 2017, 63% of the patients admitted had a rehabilitation plan of care established within 24 hours of admission. To address this, a burn order set was developed. This burn order set had the orders for PT and OT pre-populated to have the order placed on admission. The order set went live in November of 2017. In 2018, the percentage of patients receiving a comprehensive rehabilitation plan within 24 hours of admission had increased to 80%. Further education for use of the order set was provided, including additional presentations during our resident "boot camp" in 2018, our burn program manager reviewing all burn admissions and providing feedback to providers to use the burn order set, and established the role of a dedicated burn NP along with an everyday burn clinic in 2020. Staffing patterns were assessed in collaboration with the lead PT and OT. Rehabilitation established more coverage on the weekends and holidays to ensure staff was available 7 days per week to respond to the rehabilitation referrals for our patients.

**Results:**

The compliance with the ABA verification criteria for establishment of a comprehensive rehabilitation program within 24 hours of admission had increased with consistent use of the burn order set and increased rehabilitation staffing. In the last 4 quarters of 2020-2021, our compliance has increased to 92%. In 2 of the last 4 quarters our compliance was 100%.

**Conclusions:**

The use of a comprehensive burn order set can help a hybrid burn center ensure they are set up to meet ABA verification criteria. These order sets when used consistently can not only ensure consults are ordered, but also help remind providers of the unique needs of the burn patients and improve efficiency.

